# Distributed Agent-Based Orchestrator Model for Fog Computing

**DOI:** 10.3390/s22155894

**Published:** 2022-08-07

**Authors:** Agnius Liutkevičius, Nerijus Morkevičius, Algimantas Venčkauskas, Jevgenijus Toldinas

**Affiliations:** Department of Computer Science, Kaunas University of Technology, 44249 Kaunas, Lithuania

**Keywords:** fog computing, internet of things, service placement, fog service orchestration, distributed orchestrator, agent-based orchestrator

## Abstract

Fog computing is an extension of cloud computing that provides computing services closer to user end-devices at the network edge. One of the challenging topics in fog networks is the placement of tasks on fog nodes to obtain the best performance and resource usage. The process of mapping tasks for resource-constrained devices is known as the service or fog application placement problem (SPP, FAPP). The highly dynamic fog infrastructures with mobile user end-devices and constantly changing fog nodes resources (e.g., battery life, security level) require distributed/decentralized service placement (orchestration) algorithms to ensure better resilience, scalability, and optimal real-time performance. However, recently proposed service placement algorithms rarely support user end-device mobility, constantly changing the resource availability of fog nodes and the ability to recover from fog node failures at the same time. In this article, we propose a distributed agent-based orchestrator model capable of flexible service provisioning in a dynamic fog computing environment by considering the constraints on the central processing unit (CPU), memory, battery level, and security level of fog nodes. Distributing the decision-making to multiple orchestrator fog nodes instead of relying on the mapping of a single central entity helps to spread the load and increase scalability and, most importantly, resilience. The prototype system based on the proposed orchestrator model was implemented and tested with real hardware. The results show that the proposed model is efficient in terms of response latency and computational overhead, which are minimal compared to the placement algorithm itself. The research confirms that the proposed orchestrator approach is suitable for various fog network applications when scalability, mobility, and fault tolerance must be guaranteed.

## 1. Introduction

Fog computing is an extension of cloud computing that uses heterogeneous and geographically distributed fog nodes to provide services and computation closer to data-generating user end-devices, thereby reducing the amount of data forwarded to the cloud, and minimizing bandwidth requirements and request-response time. According to the OpenFog Reference Architecture for Fog Computing [1], fog computing “is a system-level horizontal architecture that distributes resources and services of computing, storage, control, and networking anywhere along the continuum from cloud to things, thereby accelerating the velocity of decision-making. Fog-centric architecture serves a specific subset of business problems that cannot be successfully implemented using only traditional cloud-based architectures or solely intelligent edge devices”.

**Figure 1:**
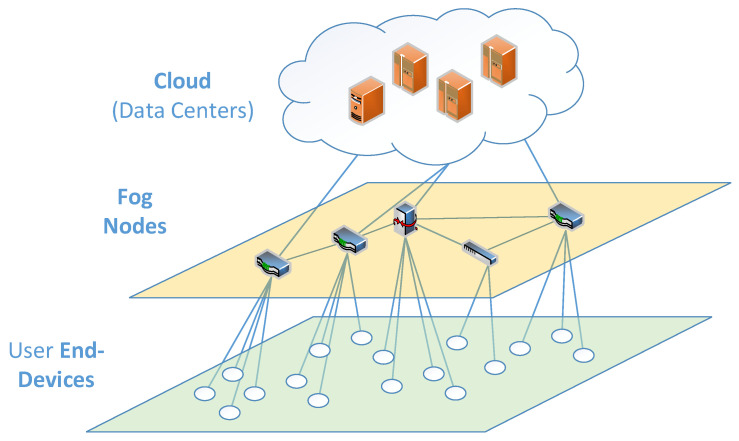
General Fog Computing architecture.

Layered architecture is the most common representation of the fog computing paradigm. Specifically, a three-layer architecture is often used to represent a fog computing infrastructure [2], as shown in Figure 1.

Although the number of fog computing layers varies in different publications, it can be seen that Internet of Things (IoT) and Cloud layers are present in all of them, while the main differences are manifested as different ways of structuring the Fog Layer [2]. As generalized in [3], the IoT layer is closest to end-users and contains various IoT devices. These devices provide various IoT applications for the end-users, including, but not limited to, agriculture [4], structural health monitoring [5], healthcare [6], vehicles [7], smart cities [8], and smart homes [9].

It is geographically distributed and produces data that are forwarded to the fog layer for processing and storage. The fog layer consists of various fog node devices that are able to process and store user requests. These devices, fixed (static) or mobile, are connected to the cloud servers and can send requests to data centers. The cloud layer consists of several data centers, which are able to perform complex computations and store a large amount of data [3].

The following six characteristics are essential in distinguishing fog computing from other computing paradigms [10]: low latency, geographic distribution, heterogeneity, interoperability and federation, real-time interactions, and scalability; wireless access and communications, as well as support for mobile user end-devices, are two additional characteristics often associated with fog networks [10]. According to the National Institute of Standards and Technology (NIST), the fog node is an essential component of the fog computing architecture [10]. Fog nodes are either physical components (e.g., gateways, switches, routers, servers, etc.) or virtual components (e.g., virtualized switches, virtual machines, cloudlets, etc.) that are tightly coupled with the smart end-devices (usually IoT) or access networks and provide computing resources to these devices [10]. In order to support the six essential characteristics of fog computing mentioned above, fog nodes need to support one or more of the following attributes: autonomy, heterogeneity, hierarchical clustering, manageability, and programmability [10]. Fog nodes usually feature limited and very heterogeneous resources, are highly geographically distributed—often mobile—and are owned and managed by various service providers [11].

### Problem and Purpose of the Study

Fog architectures should ensure that processing of data generated by end-devices is done at the most suitable fog nodes, depending on various application requirements (CPU, memory, energy, bandwidth, etc.) and fog nodes constraints. Since fog nodes are distributed and heterogeneous in nature, it is a challenge to select a fog node where the data processing application should be placed for best performance and resource usage. Therefore, fog computing requires new methodologies for fog application placement optimization, dealing with limited hardware resources and large-scale deployments, unstable connectivity and platform/operators heterogeneity, and (node or link) failures [11]. Moreover, the dynamicity of fog infrastructure with mobile end-devices and constantly changing fog nodes resources (e.g., battery life, security level) require distributed service placement (orchestration) approaches to ensure better resilience, scalability, and the best real-time performance. To achieve this goal, we propose a distributed service placement approach, which is capable of flexible service provisioning in a dynamic fog computing environment. Specifically, we propose an agent-based service orchestration model consisting of distributed decision-making agents in every fog node. The proposed solution increases scalability and adaptability and is able to cope with fog node failures, end-node mobility, and withdrawal.

This article is organized as follows. Section 2 presents the related work, Section 3 discusses the proposed orchestrator model, Section 4 covers experimental setup and the results obtained, Section 5 discusses the results, and Section 6 concludes the article.

## 2. Related Work

According to recent systematic literature reviews [2,3,11,12], the service placement problem is being solved using various architectures and techniques, which differ in terms of control plan design (centralized vs. distributed), deployment decision time (offline vs. online), support of dynamicity of the system (static vs. dynamic placement), and mobility support. As surveyed in [3], there are two common placement and management strategies: centralized and distributed (decentralized) coordination. Centralized placement algorithms have a potential advantage in finding globally optimal solutions, but are vulnerable with respect to their scalability, computational complexity, and resilience. However, most of the proposed application placement methods are centralized. In contrast, distributed placement algorithms utilize multiple coordinator/orchestrator nodes to control service mapping. This approach is more flexible, scalable, and efficient in dealing with the dynamicity of fog infrastructure but does not guarantee the global optimality of computed solutions [3].

The service placement decision can be made offline or online. The offline approach considers that all system requirements and constraints are known beforehand, while online placement decisions are made during the run-time of the system. As noticed in [3], in most real-life scenarios, service placement should be treated as an online problem that accommodates the dynamic behavior (changes) of the system.

The dynamicity of the system can be two-fold: the dynamicity of fog infrastructure and the dynamicity of applications. As described in [3], fog networks are highly dynamic with appearing and disappearing user end-nodes and fog nodes, failures and instabilities of the network links, varying capabilities of fog nodes, and changing application requirements. Therefore, the dynamic placement approach should be able to deploy new applications and remove/replace existing ones to meet changing fog infrastructure capabilities and application demands.

Finally, one of the most important features of the fog network, which should be addressed by the application placement approach, is user end-nodes and fog nodes mobility. As concluded in [3], mobility is a major challenge in fog computing since placement solutions should ensure that users always get the required services and resources without interruptions. These solutions should be able to react to the changing locations of end nodes (or fog nodes) and move services between fog nodes, following the changing position of end-devices.

### 2.1. Knowledge Gap

Despite the huge number of research papers that solve the aforementioned problems related to service and application placement, only a few of them support fully distributed, dynamic, mobility-supporting, and resilient service placement at the same time.

Some recent studies propose the dynamic placement of services, thereby supporting mobility of end-users, but use centralized or clustered (not fully distributed) approaches like Yousefpour et al. [13], Mahmud et al. [14], Velasquez et al. [15], Saurez et al. [16], Chen et al. [17], Filiposka et al. [18], Mseddi et al. [19], Josilo and Dan [20], Wang et al. [21], and Zhu et al. [22].

The other studies, including Lee et al. [23], Aral and Ovatman [24], Selimi et al. [25], Lee et al. [26], Tocze and Nadjm-Tehrani [27], Castellano et al. [28], Liu et al. [29], Alli and Alam [30], Al-Khafajiy et al. [31], Charântola et al. [32], Zhang et al. [33], and Guerrero et al. [34], investigate the application of distributed service placement solutions, but usually do not directly address resilience and fog node failures or user mobility and network dynamicity.

As concluded in the review [3], only a few approaches perform service placement in a distributed and dynamic way [16,21,24,25] and still fewer that consider distributed solutions that handle system dynamicity and support the mobility of end-users or fog nodes as proposed in [21].

To our knowledge, the most similar works include Casadei and Viroli [35], which presents a decentralized (federated) control approach, rather than fully distributed, and Jalali et al. [36], which uses machine learning methods to predict optimal placement, while our approach includes the combinatorial placement algorithm—presented in our previous work [37]. The Fog Computing for Robotics and Industrial Automation (FORA) platform, proposed by Pop et al. [38], also uses the collaboration and synchronization of fog nodes, therefore each node can make local decisions about which application tasks it will execute. In contrast to our approach, this study addresses application failures instead of fog node failures and requires fog nodes with high resource capacity.

### 2.2. Aim and Principal Conclusions

The research presented in this paper contributes to the field of resilient service placement in dynamic fog environments. We propose a distributed service placement approach and an orchestration model that includes distributed decision-making agents in every fog node, which is capable of flexible service provisioning and is resilient to fog node failures. The proposed architecture was implemented as a prototype system, which continuously monitors the incoming tasks and the state of fog nodes to make placement decisions in real-time. Unlike the majority of prior works that rely on a central control entity, we propose the distributed orchestration model using orchestrator agents in every fog node. The service placement or migration decision is made at the nearest fog node, thereby considering the state of other fog nodes. This enables proper decision making, saves energy, reduces delay, and improves the overall performance of the system. Furthermore, our proposed architecture is resilient to fog node failures by continuously monitoring the state of each fog node and migrating/restoring services to healthy fog nodes.

This work evaluates the efficiency of the proposed model and its ability to deal with constantly changing computational resources, fog node failures, end-node mobility, and withdrawal. The effectiveness of the approach is evaluated in terms of response time latency (delay) and computational overhead, which are influenced by the inter-agent communications of the distributed orchestrator.

## 3. Proposed Agent-Based Orchestrator Model

In this section, we discuss the goals of designing an agent-based distributed service placement approach and describe the architecture of the whole system.

### 3.1. Design Goals and Architecture

The design of the proposed architecture is based on three key objectives. The first objective is to improve the computational performance and power efficiency of the fog node devices by efficiently distributing tasks (services) among them. The second is to react to the rapid changes in the fog environment due to the moving user end-devices and constantly changing resources of the fog nodes, including complete failures of the fog nodes. The third is to discover the available resources of nearby fog nodes or the cloud automatically and use them to allocate tasks when required.

Each fog node can communicate with other nearby fog nodes using various types of wireless or even wired communications such as Wi-Fi, Bluetooth, ZigBee, Ethernet, etc. Fog nodes can be directly connected to the Internet and Cloud network, though this is not a necessary requirement. Furthermore, fog nodes are heterogeneous and can have different computational resources, including CPU utilization levels, memory utilization levels, battery levels (energy), and security levels.

**Figure 2:**
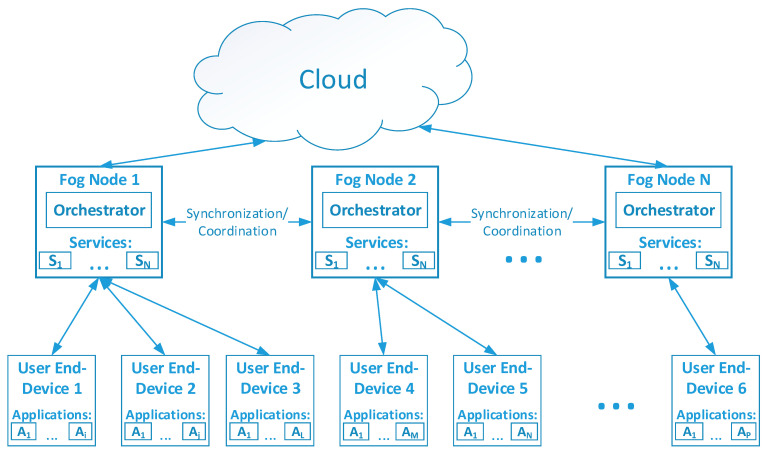
Model of the proposed distributed orchestrator architecture.

Each fog node in this system can be used to perform a set of predefined tasks (services) requested by the user end-devices, as shown in Figure 2. Therefore, fog nodes become task receivers while end-nodes are task requesters.

All fog nodes broadcast their available computational resources to their neighbors, so each node always knows how many resources are left in the remaining infrastructure. When a user end-device requests the nearest fog node to perform a task (makes a service request), the following steps are performed:

- The fog node (task receiver) identifies the type of task and the resources needed for such task.
- The fog node checks the free resources left in each neighboring fog node (including itself) that can serve the request and calculates the best placement for the requested service, using the combinatorial placement algorithm presented in our previous work [37].
- The task is then placed on one of the fog nodes by activating the appropriate service needed for the user end-device. The service might be activated at the task receiver node itself or in one of the nearby nodes or the cloud. If the service is not yet installed at the selected fog node, then it is downloaded from the cloud.

The already placed services can be relocated (activated/deactivated) to different fog nodes depending on the changes of hosting fog node computational resources, fog node failures, and the changes in the position of the user end-devices. Basically, there are few events that can initiate a new placement procedure:

- The resources of any fog node changed significantly. These can include changes in the battery level and the security level. In such a case, the fog node with depleted battery or lower than required security level (e.g., due to attack) initiates a new placement calculation procedure, and currently active services are relocated and distributed to the neighboring fog nodes to ensure system stability and resilience.
- The fog node unexpectedly fails and cannot provide any services it is currently running. This situation is detected by other fog nodes based on missing heartbeat messages from the failed node. The remaining fog nodes update their tables of available computational resources by removing resources of the failed fog node. When user end-devices will request unavailable services again (after predefined timeout), the healthy fog nodes will be aware of missing neighbor and will calculate the best placement using the remaining resources.
- The end-device moves between fog nodes, which results in disconnection from one fog node and connection to another fog node. If the end-device disconnects from the fog node, the latter detects this after some timeout, stops the unnecessary service(s), thereby freeing related resources, and informs other fog nodes about this. When the end-device requests services from the other nearby fog node on its movement path, this fog node already knows how many resources are left in the whole network and can find service placement for the new request.

**Figure 3:**
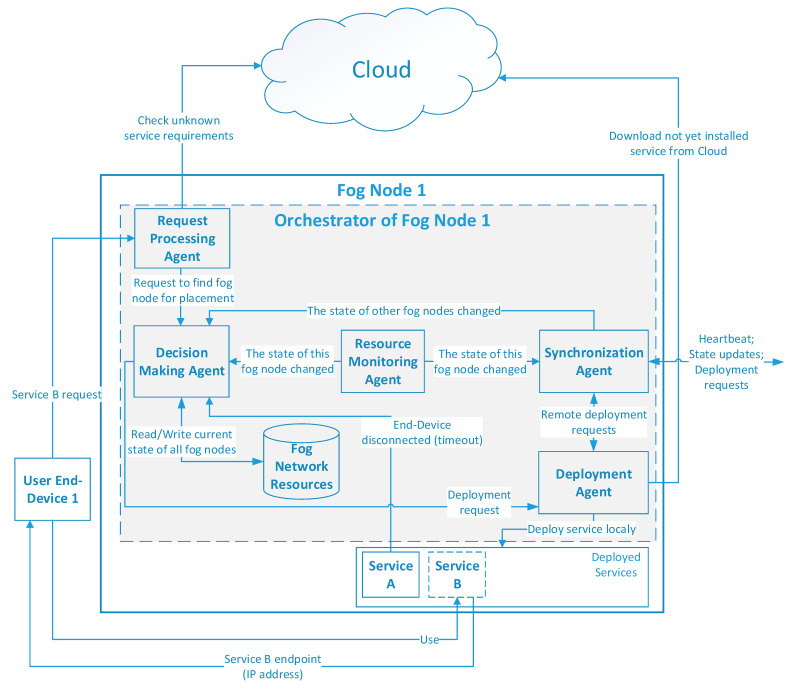
Structure of the agent-based orchestrator component of one fog node.

All aforementioned actions are performed by the Orchestrator components, which are deployed on every fog node. The orchestrator consists of software agents, including the Synchronization Agent, Decision Making Agent, Resource Monitoring Agent, Request Processing Agent, and Deployment Agent, as depicted in Figure 3.

**Figure 4:**
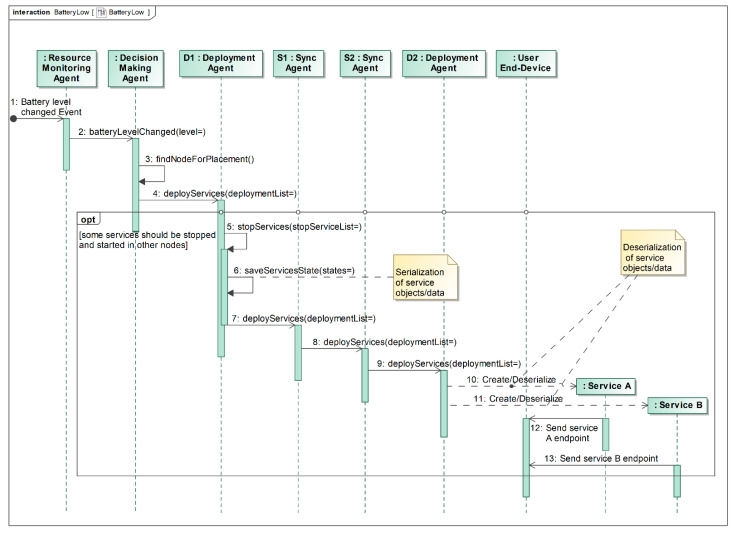
Relocation of services in case of low computational resources.

**Figure 5:**
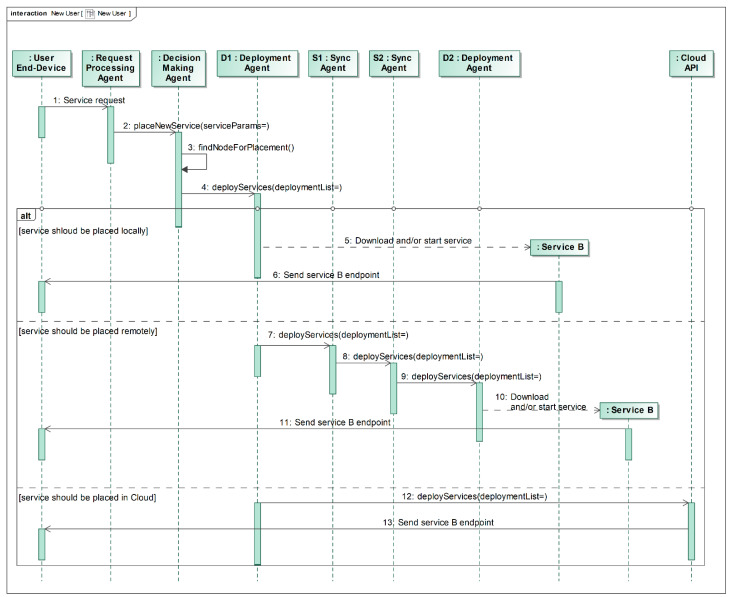
New service deployment, depending on the destination fog node given by the best placement algorithm.

The Resource Monitoring Agent constantly monitors local computational resource changes and notifies the Decision Making Agent and the Synchronization Agent if this has occurred. The Decision Making Agent stores a resource table for all fog nodes and keeps it up-to-date with notifications from the Monitoring and Synchronization Agents. When resource-related changes occur (e.g., the battery is almost depleted, see Figure 4 for details) or a request is received from the User End-Device via the Request Processing Agent (Figure 5), the Decision Making Agent calculates a new solution for service placement based on the available Fog Network Resources table.

In the case of a depleted battery, the Resource Monitoring Agent detects that the battery level changed significantly (step 1 in Figure 4) and informs the Decision Making Agent about this change (step 2 in Figure 4). The Decision Making Agent finds the new placement for the service(s), considering the remaining resources of all fog network nodes (step 3 in Figure 4), and asks the Deployment Agent to redeploy services if necessary (step 4 in Figure 4). If services need to be redeployed, the Deployment Agent stops these services, saves their states, and commands the Synchronization Agent to deploy services remotely (steps 5, 6 and 7 in Figure 4). The Synchronization Agent communicates with the appropriate synchronization agent(s) of the other fog node(s) and asks to start new services (step 8 in Figure 4). Finally, the remote Deployment Agent(s) fulfills the request by starting and restoring the required services (steps 10 and 11 in Figure 4), and the User End-Device is provided with new connection endpoints (steps 12 and 13 in Figure 4).

When the User End-Device makes a service request (e.g., asks to provide service B, step 1 in Figure 5), the Request Processing Agent checks the computational requirements for this service and asks the Decision Making Agent to find the best service placement (activation) solution (step 2 in Figure 5). The Decision Making Agent finds the best fog node to deploy that service (step 3 in Figure 5) among all fog nodes to which the End-Device can connect (including the local fog node). Here, we have three possible cases (‘alt’ region in Figure 5):

- The service should be deployed locally. Then, the Decision Making Agent requests the Deployment Agent to download the service (step 4 in Figure 5) and install it or just activate this service if it is already installed (steps 5 and 6 in Figure 5).
- The service should be deployed on the neighboring node. The Decision Making Agent requests the Deployment Agent to install the service remotely. The local Deployment Agent notifies the destination fog node through the Synchronization Agent to install the service (steps 7, 8 and 9 in Figure 5) and the remote Deployment Agent does so on the remote node (steps 10 and 11 in Figure 5).
- The service cannot be deployed on any fog node due to lack of resources (steps 12 and 13 in Figure 5). In such a case, the service is deployed in the cloud (which is treated as a node with very high resource capacity).

**Figure 6:**
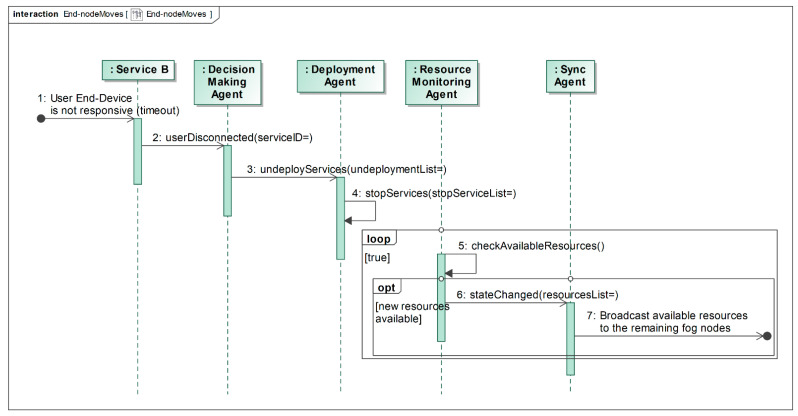
Disconnection from the fog node, when the user end-device moves between fog nodes.

When the User End-Device moves between fog nodes, it disconnects from the current fog node and tries to connect to other, more adjacent fog nodes (with higher signal strength). The disconnection is detected by the service(s), currently provided to the End-Device (step 1 in Figure 6). After some predefined timeout (connection attempts), each service informs the Decision Making Agent that the User End-Device is disconnected (step 2 in Figure 6). The Decision Making Agent updates its computational resources list and asks the Deployment Agent to stop unnecessary services and free resources (step 3 and 4 in Figure 6). The Resource Monitoring Agent detects that the resources are freed (step 5 in Figure 6) and informs other fog nodes via the Synchronization Agent (steps 6 and 7 in Figure 6). The remaining algorithm is exactly the same as presented in Figure 5: the User End-Device requests service(s) from the other nearby fog node on its movement path, which is already notified about the new resource distribution in the entire network and can calculate the service placement for the new request.

Regardless of whether the service is deployed locally or remotely, it notifies the User’s End-Device by sending the IP address that should be used to access the service.

**Figure 7:**
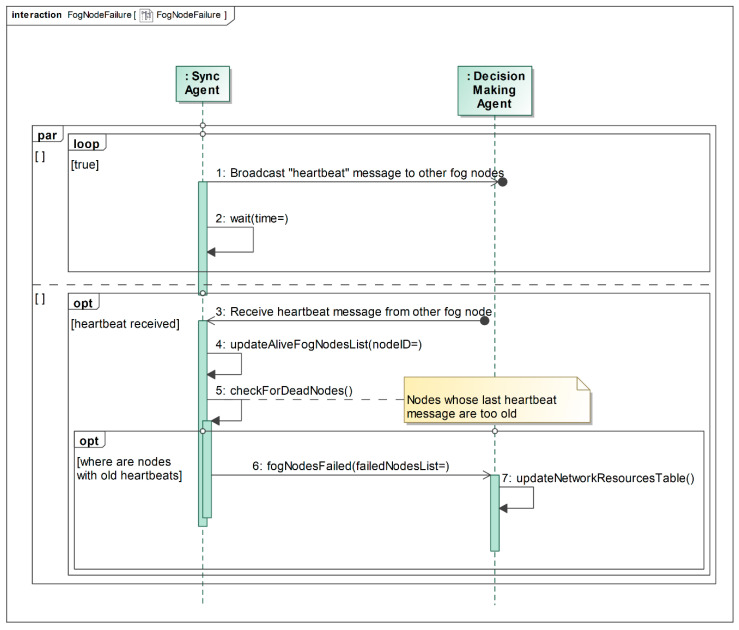
Fog node failure detection using heartbeat messages.

In the case of fog node failure, the Synchronization Agents of other fog nodes detect this situation by checking the last heartbeat messages from the failed fog node, as depicted in Figure 7. Synchronization Agents regularly broadcast heartbeat messages (the infinite loop in Figure 7). At the same time, when the Synchronization Agent receives a heartbeat message from the other fog node, it updates the list of alive fog nodes (step 4 in Figure 7) and then checks if there are old heartbeat timestamps (step 5 in Figure 7). If there are any dead nodes with very old heartbeat timestamps, the Synchronization Agent informs the Decision Making Agent by sending the list with the failed fog nodes (step 6 in Figure 7). The Decision Making Agent updates the network resources table and removes the inactive fog nodes from it (step 7 in Figure 7). The heartbeat period (*wait()* function parameter in Figure 7) and the age of the “good” heartbeat are configurable parameters and can be adapted for various scenarios and applications. Finally, when a fog node fails, the User End-Devices request services from other healthy fog nodes, which already know about the failure and do not take into account the resources of the lost fog node while calculating service placement.

All coordination between fog nodes is performed by the Synchronization Agents, which:

- broadcast the changes in computational resource availability of the fog nodes;
- broadcast the heartbeat messages (when the node stops this broadcast, other Synchronization Agents remove the node from the Fog Network Resources list);
- send remote deployment requests when needed.

### 3.2. Properties That Affect Service Placement

The service placement algorithm considers the properties and constraints of all the components of the fog network to make the appropriate service placement decision. These include node properties, network properties, service (task) properties, and user application requirements.

**(1) Node properties:**

Each fog node in our model at any given time can have different capacities of computational resources, which include a CPU utilization level from 1–100%, a random access memory (RAM) utilization level from 1–100%, a remaining battery level from 1–100%, and a level of security from 1–3, which describes how strong the data and communications protected in a particular fog node are.

**(2) Network properties:**

The service placement decision must take into account the ability of the end-user device to connect to the fog node(s) where the service(s) will be placed. Since fog nodes are distributed for better coverage of the area, the placement algorithm does not consider fog nodes that are too far away from the user end-device. This constraint reduces the complexity of the placement calculations and decreases latency.

**(3) Service properties:**

Each service demands some resources, provided by fog nodes: CPU, RAM, and security level. The service requirements are predefined and each fog node knows these requirements for each possible service. If the user end-device requests an unknown service, the fog node uses cloud services to get all service-related data.

The service placement algorithm must ensure that the service that is being deployed will have enough resources and its deployment will not negatively affect already deployed services.

### 3.3. Service Placement Optimization Method

The service placement optimization method used in this research was proposed in [37]. This multi-objective optimization method is suitable for finding the best placement of *n* available services in *k* fog nodes according to the given constraints and conditions. In this method, the QoS parameters of the i-th possible placement $X_{i}$ are expressed by the values of the objective $f_{j}\left(X_{i}\right)$￼j=1,2,…,m￼ and constraints are given by equations:

(1) $$\{\begin{array}{c} g_{j}\left(X_{i}\right)\geq 0,j=1,2,\ldots ,n_{g}\\ h_{k}\left(X_{i}\right)=0,k=1,2,\ldots ,n_{h} \end{array}.$$

The main objective of the optimization process is to find the service placement $X_{opt}$ which minimizes all the objective functions $f_{j}$:

(2) $$X_{opt}=arg\underset{i}{min}\;F\left(X_{i}\right).$$

The objective functions may include the overall security of the whole system $f_{sec}\left(.\right)$, CPU usage $f_{CPU}\left(.\right)$, RAM usage $f_{RAM}\left(.\right)$, network bandwidth usage $f_{BW}\left(.\right)$, power usage $f_{PW}\left(.\right)$, energy usage $f_{EN}\left(.\right)$, etc. (descriptions and a more detailed discussion on the construction of suitable objective functions are presented in [37]).

**Figure 8:**
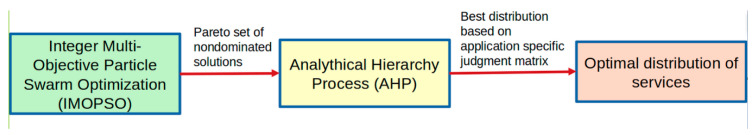
Flow chart of the service placement finding method.

The objective functions contradict each other, and usually there is no single solution that minimizes all the objective functions at the same time. To solve this problem, the two-stage optimization process presented in Figure 8 is used.

The Integer Multi-Objective Particle Swarm Optimization (IMOPSO) process is used to find a Pareto set of solutions to the problem. The members of this set are non-dominated service placements, meaning that each of them is better than all the other placements by at least one objective function.

The second step uses the Analytical Hierarchy Process (AHP) [39] to choose the best solution from the Pareto set. AHP employs only pairwise comparisons of all alternatives for all objective functions. AHP uses the so-called judgment matrix, which is application specific and represents the importance of the objective functions in the specific application area.

The pseudo code of the IMOPSO method is presented below:

1. Generate r random particles: $X_{i}={\left(x_{1}^{i},x_{2}^{i},\ldots ,x_{n}^{i}\right)}^{T}$, i=1,2,…,r.
2. Initialize Pareto set: R=∅.
3. Assign the initial velocities $V_{i}=\vec{0}$, i=1,2,…,r.
4. Initialize the global best position and the global best score: $gBPos=\vec{0}$, $gBPos=\vec{inf}$.
5. Assign the initial best scores and best positions to all particles: $pBest_{i}=\vec{inf}$, $pBPos_{i}=X_{i}$, i=1,2,…,r.
6. With each particle $X_{i}$ (i=1,2,…,r) do:
  a. Calculate the values of each objective function: $F_{i}={\left(f_{1}\left(X_{i}\right),f_{2}\left(X_{i}\right),\;\ldots ,f_{m}\left(X_{i}\right)\right)}^{T}$.
  b. If $F_{i}$ dominates $pBest_{i}$: $pBest_{i}=F_{i}$, $pBPos_{i}$=$X_{i}$, $X_{i}\to R$.
  c. If $F_{i}$ neither dominates nor is dominated by $pBest_{i}$: with probability 0.5 do: $pBest_{i}=F_{i}$, $pBPos_{i}$=$X_{i}$, $X_{i}\to R$.
  d. If $F_{i}$ dominates gBest: $gBest=F_{i}$, gBPos=$X_{i}$.
  e. If $F_{i}$ neither dominates nor is dominated by gBest: with probability 0.5 do: $gBest=F_{i}$, gBPos=$X_{i}$.
  f. Calculate new velocity: $V_{i}=wV_{i}+r_{1}\left(pBPos_{i}-X_{i}\right)+r_{2}\left(gBPos-X_{i}\right)$.
  g. Update the position of the particle: $X_{i}=round\left(X_{i}+V_{i}\right)$.
  h. If the particle is out of the range: $V_{i}=-V_{i}$, $X_{i}=X_{edge}$.
7. Analyze set R and remove all the duplicated and dominated entries.
8. If maximum number of iterations is not reached go to step 6.
9. The set R contains Pareto frontier solutions.

These are the meanings of the main notations: *n*—total number of services; *k*—total number of available fog nodes; *m*—total number of evaluation criteria (objective functions); *r*—number of particles used in IMOPSO algorithm; $X_{i}$—the i-th possible distribution of *n* services among *k* fog nodes (position of the i-th particle in n-dimensional definition area); $x_{j}^{i}$—is the j-th element of vector $X_{i}$, the meaning of this element’s value is that the j-th service must be placed in the $x_{j}^{i}$-th fog node (for example, vector $X_{1}={\left(2,\;3,2,1\right)}^{T}$ means that the first service is placed into the second fog node, the second service is placed into the third fog node, etc.); $V_{i}$—velocity of the i-th particle; $f_{i}\left(x\right)—i$-th objective function (optimization criteria); $F_{i}$—score vector of the i-th particle; $X_{edge}$—nearest particle position on the edge of the definition area; R—a set of Pareto frontier solutions; $pBest_{i}$—best score of the i-th particle; $pBPos_{i}$—best position of the i-th particle; gBest—best global score of all the particles; and gBPos—position of the particle with the best global score.

The pseudo code of the AHP process is presented below:

1. Load a m×m judgment matrix $Q=\left(q_{i,j}\right)$, i,j=1, 2, …,m with the application specific results of criteria $f_{k}\left(.\right)$ (k=1,2,…,m) pairwise comparisons.
2. With each criterion (objective function) $f_{k}\left(.\right)$, k=1,2,…,m do:
  - Construct the weight coefficient matrix $W_{k}=\left(w_{i,j}\right)$: $w_{i,j}=comp_{k}\left(X_{i},X_{j}\right)$, i,j=1, 2, …, s, s=|R| using all the alternatives from set R.
3. Use AHP decision-making method and get alternative priority vector P, $P={\left(p_{i}\right)}^{T}$, i=1, 2, …,m: $P=AHP(Q,W_{k}$), k=1,2,…,m.
4. Choose the best service distribution alternative $X_{opt}$, which corresponds to the highest priority $p_{opt}=max\left(p_{i}\right)$￼i=1, 2, …,m￼ as the final solution of an optimization process.

The main notations used in this pseudo code are: Q—the judgment matrix with results of pairwise criteria comparison; $comp_{k}$—function of pairwise comparison of two possible service distributions; $W_{k}$—weight coefficient matrix with pairwise comparisons of all distributions of the services from the set *R*; P—priority vector of alternatives (vector element with highest value $p_{opt}$ corresponds to the best alternative); $X_{opt}$—best service distribution alternative.

A more detailed description of the service placement optimization algorithm with an explanation of the meanings of all notations is presented in [37]. In this research, we used the method for implementation of the Decisions Making Agent and evaluated its performance inside the agent-based orchestrator operating in the fog nodes.

## 4. Experimental Setup and Results

### 4.1. Experimental Setup

We used a two-fold approach to evaluate the properties of the proposed agent-based orchestrator model. Real hardware was used to evaluate the real-world characteristics of the building blocks of the proposed model, i.e., the performance of the communication protocols, capabilities of the underlying hardware, etc., was measured using prototype hardware sensors and actuators. On the other hand, it is very difficult to use real hardware while evaluating the scaling properties of the model and trying to reproduce results in order to repeat the exact same experiments with different parameters. To achieve repeatability, we implemented the proposed model as a multi-agent system in which all agents act exactly the same as they would in the real system, but they are not interacting with real hardware. In this system, external events, measurements of environment parameters, and activations of end devices are emulated by special agents. All the performance characteristics emulated during the experiment were measured using real hardware devices in order to bring the overall model performance evaluation results as close as possible to the reality.

**Figure 9:**
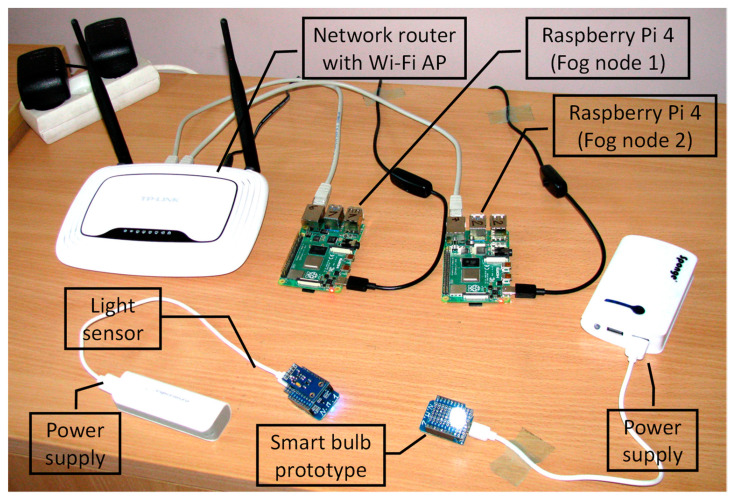
Hardware setup used for the experimental evaluation of the proposed model.

The hardware setup used for the experimental evaluation is presented in Figure 9.

Two fog nodes were implemented using the Raspberry Pi 4 Model B computers with 4 GB of RAM. They were running Raspbian 10 OS and JDK 1.8.0_333. The multi-agent system was implemented using the Jade 4.5 library. To evaluate the scalability of the proposed model in some experiments, the role of fog nodes was performed by a personal computer equipped with Intel Core i5-4570 CPU with 16 GB of RAM and running Windows 10 Pro OS with JDK 1.8.0_333. All fog nodes were connected to the local area network (LAN) using a gigabit Ethernet switch integrated into the wireless router. The prototypes of the end-devices were implemented using ESP8266 microcontrollers, which used integrated Wi-Fi capabilities to connect directly to the same wireless router. All communications between sensors and actuators are performed using the TCP/IP protocol and through the corresponding agents running inside the fog nodes.

### 4.2. Results

The main objective of the experimental evaluation was to find out how the nature of the multi-agent system, i.e., lots of small agents performing single dedicated roles and communicating with each other, may impact the overall performance of the proposed model. Two scenarios, described in Figure 4 and Figure 5, were implemented, which include service relocation and new service deployment.

**Figure 10:**
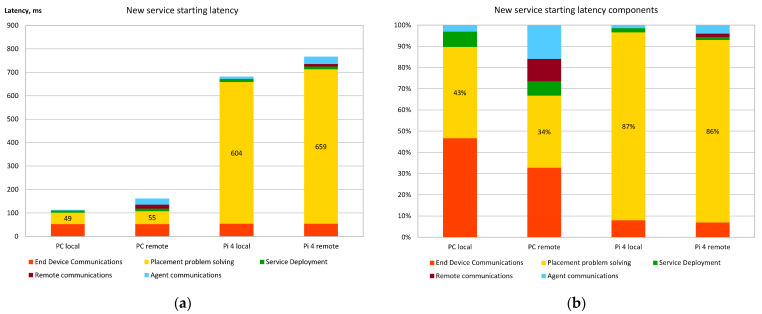
Evaluation of the latency of the new service starting: (**a**) Absolute latency; (**b**) Relative latency caused by different parts of the placement algorithm.

Figure 10 shows the latency evaluation of the new service deployment/start. Two alternatives of this scenario were performed using Raspberry Pi 4 (shown in Figure 10 as “Pi 4”) and personal computers (PC) as fog nodes. The first alternative deploys the requested services on the same fog node as the Request Processing Agent is running (shown as “local” in Figure 10). The second alternative deploys the required services on the different fog nodes, thus requiring additional communications between Synchronization and Deployment Agents. In this scenario, an additional 11 services were running, thus making the service placement problem solved by the Decision Making Agent more close to the real conditions.

To register the latency of the agent-based system, a special GlobalTimer class was developed. This class uses the System.nanoTime() method and stores the time and additional related information on the events. To reduce the impact of the log subsystem on the system performance, all log information is stored only in RAM and printed to the console only after the explicit request. The logging class can provide reports of all recorded events grouped by the involved agent’s name, the instance of related event, and the system time. Each agent in the system uses the same instance of GlobalTimer class and logs all significant events, such as creation and death of the agent, times when each inter-agent communication message was received and sent, and times when the important cycles of agent life (e.g., start/finish of agent migrations, start/finish of placement problem solving process, etc.) were started and finished. Using the logging object, it is possible to view how one concrete event was processed by the agents, which agents were involved in the process, how long it took to perform various tasks inside the agents, etc.

Figure 10a shows the full time taken by the whole process, starting with the request from the end-device and ending when the required services are actually running in the appropriate fog node. Figure 10b shows which parts of the multi-agent orchestrator model have the biggest impact on the overall latency.

**Figure 11:**
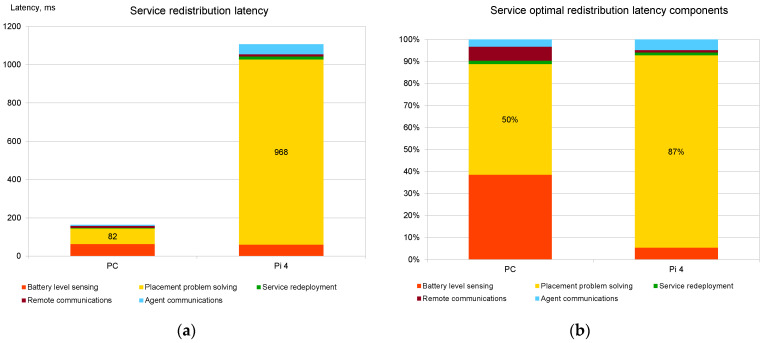
Evaluation of service redistribution latency: (**a**) Absolute latency; (**b**) Relative latency caused by different parts of the placement algorithm.

Figure 11 shows the evaluation of the latency of the scenario described in Figure 4. In this case, the low-battery event was emulated, and the new service placement process was initiated. The parameters were chosen in such a way that three services from fog node No. 1 needed to be moved to fog node No. 2. An additional 9 services were used to make the service placement problem solved by the Decision Making Agent more realistic. Figure 11a shows the overall time taken from the generation of a low-battery event until all three services are started on the corresponding fog node. Figure 11b describes the impact of the various parts of the multi-agent system.

The evaluation of the results clearly shows the following tendencies:

- Agent communications within fog nodes and simple decisions made by them (marked as “Agent communications” in Figure 10 and Figure 11) do not add significant latency to the overall latency of the model. Figure 10b and Figure 11b show that the agent intercommunication component of the relative latency becomes significant (and takes up to 11% of the overall time) only when the fog node hardware is powerful and the service placement problem (i.e., the structure of the fog infrastructure) is relatively simple.
- Agent communications outside the local fog nodes (marked as “Remote communications” in Figure 10 and Figure 11) also do not add a very significant latency impact. Thus, there is no real basis to prefer local service deployments vs. remote ones if the proposed model is used.
- The greatest impact on latency is caused by the service placement algorithm. This issue is more relevant when relatively weak hardware is used for fog nodes.

Additional experiments were carried out to determine the performance characteristics of the service placement solving algorithm, as it has the greatest impact on the overall performance of the agent-based orchestrator architecture. The service placement finding method was fully implemented in Java and integrated into the Decision Making Agent. To make the service placement problem more deterministic, we used a set of four (*m* = 4) *n*-dimensional paraboloids as the objective functions: $f_{j}\left(X\right)=f\left(X-X_{j}^{0}\right)$ where $X={\left(x_{1},x_{2},\ldots ,x_{n}\right)}^{T}$, and vectors $X_{j\;}^{0}={\left(x_{1}^{j0},x_{2}^{j0},\ldots ,x_{n}^{j0}\right)}^{T}$, j=1, 2, …,4 are the vertices of the corresponding *n*-dimensional paraboloids, i.e., $f\left(X-X_{j}^{0}\right)={\left(x_{1}-x_{1}^{j0}\right)}^{2}+{\left(x_{2}-x_{2}^{j0}\right)}^{2}+\ldots +{\left(x_{n}-x_{n}^{j0}\right)}^{2}$. The paraboloids were chosen because we needed objective functions that can be easily generalized to *n*-dimensions, all the properties are known, the minima point is at their vertices, and they are easy enough to calculate, which corresponds to the real objective functions (for example, the objective function of RAM usage is a simple sum of RAM usages of all services placed in a fog node). To make the Pareto set not trivial in each experiment, depending on the dimensions of the area of the definition of the problem, different vertices were used for each objective function, i.e., $X_{i\;}^{0}\neq X_{j\;}^{0}$, if i≠j.

The judgment matrix was chosen in such a way that one of the objectives was of very high importance compared to the other ones (consistency ratio of the matrix is 4.5%):

$$Q=\left(\begin{array}{cccc} 1 & 7 & 7 & 7\\ 1/7 & 1 & 2 & 2\\ 1/7 & 1/2 & 1 & 2\\ 1/7 & 1/2 & 1/2 & 1 \end{array}\right)$$

**Figure 12:**
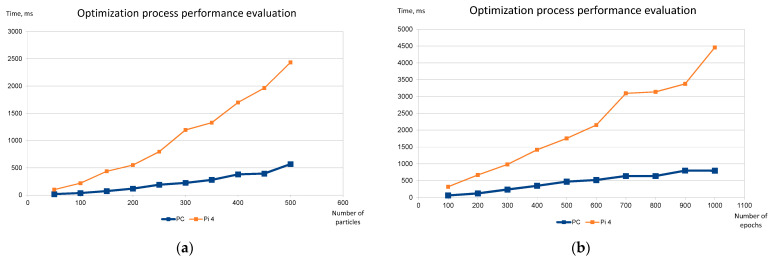
Evaluation of the service placement optimization process: (**a**) Time dependency on the number of particles (200 epochs used); (**b**) Time dependency on the number of epochs (200 initial particles used).

Therefore, it is possible to predict the result of the optimization process and to be able to evaluate the accuracy of the optimization results. The IMOPSO method uses initial random places for all particles, so the optimization process may slightly vary in performance each time. We used 20 attempts for each set of parameters and calculated the average performance values. Figure 12 shows the dependencies of the overall time taken to solve the placement problem on the number of particles and epochs used. The experiments were carried out using the Raspberry Pi 4 computer (marked “Pi 4” in Figure 12) and a personal computer (marked “PC” in Figure 12). The 12 services’ placement into 4 fog nodes problem was used. The charts show the time dependency on the initial number of particles (Figure 12a) and the total number of epochs (Figure 12b). In both cases, the AHP part of the optimization process was performed in full.

**Figure 13:**
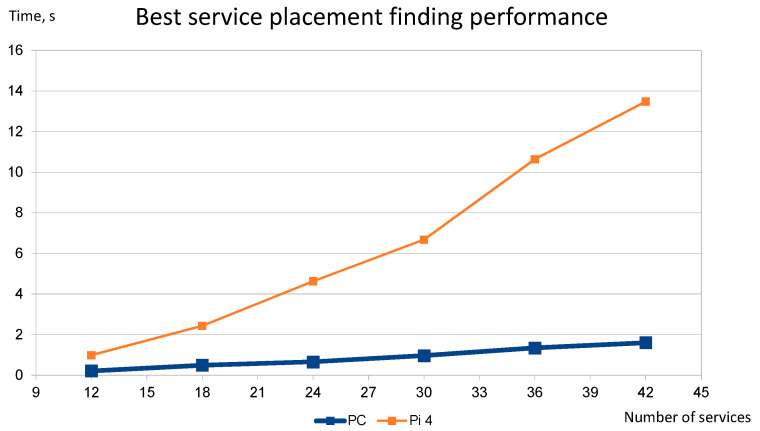
Scalability of the service placement finding algorithm.

Figure 13 shows how the service placement solution algorithm performs while solving problems of increasing complexity. The parameters used in each experiment in Figure 13 are summarized in Table 1. The values of these parameters were obtained experimentally and show sufficient accuracy of the service placement finding results (less than 5% of the placement inconsistencies). Each point in the plot was obtained by averaging the results of 20 experiments performed on the Raspberry Pi 4 and PC.

The most significant observations emerging from these experiments are the following:

- The computational time of the service placement optimization method has a linear dependence on the initial number of particles and the number of epochs.
- The computational time of the service placement optimization method has a linear dependence on the complexity of the problem in terms of the number of fog nodes and services. The IMOPSO part of the optimization process is linear by design, and the experimental results confirm that the AHP part also scales linearly with the increase in the complexity of the problem. This trend was observed by finding the recommended parameters of the optimization process for each step of complexity of the problem (summarized in Table 1). These parameters are a good starting point for using the placement finding method in real-life scenarios, as it is impossible to formally evaluate the precision of the results achieved by the optimization process.
- The total time taken by the algorithm to find the placement of the service on potential fog node hardware of “average performance” (Raspberry Pi 4 in our case) shows that it is not feasible to use this method to solve problems with more than a few tens of services.

## 5. Discussion

The experimental evaluation of the prototype system, based on the proposed distributed orchestrator model, shows low latency and small computational overhead, which are caused by the synchronization and communication part of the orchestrator algorithm. Even the Raspberry Pi 4-based fog nodes are capable of quickly deploying or redeploying the needed services once the placement calculations are made by the placement optimization algorithm. For example, the delay caused by agent intercommunications, stopping of three services, and starting of these services in a new fog node takes less than 55 ms on Raspberry Pi 4 computers. Moreover, this time is very little dependent on CPU and RAM capabilities of fog nodes, as very similar results are achieved when using a more capable PC.

The results of the study show that the selected service placement algorithm generates the highest computational overhead (up to 87% of the overall delay when used on Raspberry Pi 4), which is even greater when the number of deployed services and the number of suitable-for-deployment fog nodes are high. However, experimental results show that if an average PC is used for computations, then the placement problem with up to 30 services and 10 fog nodes is solved in less than 1 s, which is fully acceptable in real-life conditions. Moreover, the optimization algorithm scales linearly with increasing complexity of the situation, making it possible to use it in more complex situations with more powerful hardware.

However, in real conditions, the numbers of services and fog nodes are quite low, since fog nodes are distributed evenly and widely to cover a larger area, and user-demanded services can be deployed only in the few neighboring nodes. Even if this is not the case, our proposed orchestrator model is suitable for incorporating any kind of optimization algorithm, which improves the overall performance even more.

The experimental results show that even using the fully distributed orchestrator approach, the performance of the proposed system is in line with the proposals, based on the centralized or federated approach. Furthermore, the resilience in our model is much higher because any fog node can make service placement decisions. This means that the system is fully resilient to any fog node failure regardless of the number of healthy fog nodes. In contrast, federated (e.g., [35]) and especially centralized (e.g., [22]) approaches have one or several points of failure (placement decision-making nodes) that make the whole system inoperative in case they become unavailable.

The main advantage of the proposed distributed orchestrator model is its resilience to fog node failures and support for user mobility (through redistribution of the services) and ability to adapt to the constantly changing computational resources of fog nodes (through constant resource monitoring and synchronization between fog nodes) with minimal computational overhead. Our model could be applied to a wide range of low-intensity IoT applications with mobile users and no hard real-time constraints, e.g., person-oriented applications using body array networks (BAN) and personal area networks (PAN), smart home and environments monitoring and control applications, etc.

In order to apply the proposed model for larger fog networks with more strict response requirements, future studies should aim to select a faster service placement optimization algorithm, of which complexity plays a major role in the overall placement latency.

## 6. Conclusions

This paper proposes a distributed agent-based orchestrator model for fog computing. This model is based on orchestrator agents, which are distributed across all fog nodes and make service placement decisions, considering the CPU, memory, battery, and security constraints of fog nodes and the requirements of user applications.

The major contribution of our work is the novel orchestration model, which, unlike the majority of prior works, does not rely on a central control entity. Since there is no central decision-making entity, service(s) placement decision is made by the closest to the user orchestrator agent, which knows how many resources are left in the fog network via agent synchronization and monitoring mechanism. Such an approach enables scalability and resilience to fog node failures, as well as supporting the mobility of the end-users and adaptability to the constantly changing computational resources (e.g., battery level).

This work also revealed that the greatest impact on performance is the efficiency of the service placement optimization algorithm, while the communication and synchronization overhead plays only an insignificant role. Therefore, more research should focus on finding a more efficient optimization algorithm for service placement.

## Figures and Tables

**Table 1:** Optimization parameters used for the evaluation of the service placement finding algorithm.

Fog Nodes	Services	Particles	Epochs
4	12	200	300
6	18	300	300
8	24	400	400
10	30	500	600
12	36	600	700
14	42	600	800

## Data Availability

Not applicable.
